# Imaging Diagnostic and Pathology in the Management of Oncological-Patients

**DOI:** 10.1155/2019/2513680

**Published:** 2019-06-16

**Authors:** Elena Bonanno, Nicola Toschi, Alessandro Bombonati, Pietro Muto, Orazio Schillaci

**Affiliations:** ^1^Anatomic Pathology, Department of Experimental Medicine, University of Rome “Tor Vergata”, Via Montpellier 1, 00133 Rome, Italy; ^2^“Diagnostica Medica” and “Villa Dei Platani”, Avellino, Italy; ^3^Department of Biomedicine and Prevention, University of Rome “Tor Vergata”, Via Montpellier 1, 00133 Rome, Italy; ^4^Martinos Center for Biomedical Imaging, Boston, MA, USA; ^5^Harvard Medical School, Boston, MA, USA; ^6^Department of Pathology and Laboratory Medicine, Einstein Medical Center, Philadelphia, PA, USA; ^7^Radiation Oncology, Istituto Nazionale Tumori-IRCCS-G. Pascale Foundation, Naples, Italy; ^8^IRCCS Neuromed, Pozzilli, IS, Italy

Personalized medicine is one of the main objectives of both basic and translational cancer research. Nevertheless, it has become clear that the creation of personalized therapeutic protocols requires synergistic, transdisciplinary competencies. Indeed, novel approved therapies rarely take into account both the interindividual variability and the aptitude of cancer cells to undergo those genetic and molecular adaptation involved in the drug resistance phenomenon. In spite of recent and promising biomedical and biomarker discoveries, individually tailored medical care is still far from reality, and molecules which are output by preclinical trials are very rarely translatable to evaluation for the diagnostic or therapeutical potential. The discrepancy between experimental data on new anticancer molecules and the opportunity to actually employ them in both diagnosis and therapy is due to multiple factors such as biological differences between human diseases and animal models, inconsistence of experimental plans, and/or incorrect interpretation of experimental results. For example, several preclinical studies lack data validation performed by pathologists with long-term experience in cancer animal models. In view of the above, it appears evident that working towards personalized medicine in oncology requires the synergic combination of several disciplines such as nuclear medicine and anatomic pathology, which represent two complementary approaches to diagnosis, prognosis, and evaluation of therapeutic response.

The focus of this special issue is the alliance between Imaging Diagnostic (i.e., Nuclear Medicine and Radiology) and Anatomic Pathology, in the belief that a structured collaboration model between these disciplines can speed up the achievement of a medical paradigm that takes into account the uniqueness of every human being.

Out of a total of nineteen submissions, after two rounds of rigorous review, fifteen contributions were accepted for publications. Among them, four papers are focused on the breast cancer, four on lung cancer, three on prostate cancer, and three on the early detection of lymphoma. In addition, in several studies, the authors reported artificial intelligence applications for the diagnosis of tumor lesions; for example, a deep neural network architecture is able to discriminate malignant breast cancer lesions in mammographic images.

In conclusion, we felt that the novelties highlighted in this special issue can provide the scientific rationale for further investigations in translational medicine based on the combination between Pathology and Diagnostic Imaging data.

